# Prevalence of *Borrelia burgdorferi *sensu lato in rodents from Gansu, northwestern China

**DOI:** 10.1186/1471-2180-10-157

**Published:** 2010-05-28

**Authors:** Fang Zhang, Zhanwei Gong, Jijun Zhang, Zengjia Liu

**Affiliations:** 1 The Center for Disease Control and Prevention of Lanzhou Command, PLA, 509 Dong-Gang Dong Road, Chenguan District, Lanzhou 730020, PR China

## Abstract

**Background:**

Lyme disease is a multi-organ infection disease caused by *Borrelia burgdorferi *sensu lato. Lyme disease was first documented in north-east China in 1986. Since then more than 20 provinces in China were confirmed the existence of nature foci of Lyme disease. In the present study, a molecular epidemiological survey was conducted to investigate the presence of *Borrelia burgdorferi *sensu lato in rodents from Gansu Province for the first time.

**Result:**

A total of 140 rodents of 7 species were examined for *Borrelia burgdorferi *sensu lato. by nested-PCR and culture isolation. The overall infection rate was 22.86%. Two rodent species most frequently trapped were responsible for all positive. 3 strains were isolated from *Apodemus agrarius*, which belonged to *B. garinii*, 1 strain isolated from *Rattus losea *was identified as *B. afzelii*.

**Conclusion:**

The study firstly showed the role of rodents in maintaining the pathogen of Lyme disease in the environment from Gansu Province and there existed at least two genotypes of Lyme disease spirochaetes in rodents.

## Background

Lyme disease is a multisystemic zoonotic disease caused by *Borrelia burgdorferi *sensu lato (*B. burgdorferi *s. l.). *B. burgdorferi *s. l. circulates in an enzootic cycle between the primary vertebrate reservoir and the ticks[1,2]. A wide range of mammals are severeded as reservoir hosts in the natural cycle of *B. burgdorferi *sensu lato[3,4]. Different species of rodents, mainly mice and voles, are identified to be efficient natural reservoirs for *B. burgdorferi *sensu lato. They could naturally infect *B. burgdorferi *sensu lato and remain infective for a long time.

In China Lyme disease was first identified in 1986 from Heilongjiang Province [5], since then more than 20 provinces were confirmed the existence of nature foci of Lyme disease [6]. In China at least 9 tick species have been identified as the vector of *B. burgdorferi *s.l. and it also confirmed the difference of vector species varied with the geographical origin [7]. However, less is known about the prevalence and distribution of *B. burgdorferi *s.l. in rodents. Limited studies have been conducted to investigate the prevalence of *B. burgdorferi *s.l. among rodents from northwestern China [8], systemic surveys on rodents are still lacking. The objective of the study was to investigate the prevalence of *B. burgdorferi *s.l. in rodents from Gansu Province of northwestern China.

## Results

### Prevalence of *B. burgdorferi *s.l. in rodents

A total of 140 rodents of 7 species, including *Apodemus agrarius*, *Rattus losea*, *Apodemus sylvaticus*, *Rattus norvegicus*, *Mus musculus*, *Ochotoma alpine *and *Marmota himalayana *were collected and tested in the study (Table 1). *Apodemus agrarius *(*A. agrarius*) was the most frequently trapped species (85.71%) in the study sample. Out of 140 rodents examined, *B. burgdorferi *sensu lato DNA was detected in 32 rodent samples. The overall infection rate was 22.86%. *Apodemus agrarius *(*A. agrarius*) and *Rattus losea *(*R. losea*) were responsible for all positive for *B. burgdorferi *s.l.. There was no significant difference in infection rate among the 7 rodent species, although the positive rate of *B. burgdorferi *s.l. in *R. losea *was 40.0%.

**Table 1:** Results of detection and isolation for *B. burgdorferi *s.l. in rodents by species in Gansu.

Rodent species	No. of rodent tested	No.positive	No. isolate	NO. isolates for	
				***B. garinii***	***B. afzelii***

*Apodemus agrarius*	120	28	3	3	
*Rattus losea*	10	4	1		1
*Apodemus sylvaticus*	4	0	0		
*Rattus norvegicus*	2	0	0		
*Mus musculus*	2	0	0		
*Ochotoma alpine*	1	0	0		
*Marmota himalayana*	1	0	0		

Total	140	32	4	3	1

No: number

### The isolation and identification of isolates from rodents

We made an effort to isolate bacteria from all 140 rodent samples. However, spirochetes were not isolated from other samples except for from 4 PCR-positive samples. A total of 4 isolates were obtained, among which 3 isolated from *A. agrarius*: two from adult rodents, named ZGS01 and ZGS02, one from immature rodent, named ZGS03. The other one isolate from *R. losea *(Table 1) named ZGS04. All four culture isolates reacted with monoclonal antibody (H5332) by indirect immunofluorescence (IFA) with the titers ranging from 1:32 to 1:1024. On the basis of *MseI *RFLP analysis, 3 strains isolated from *A. agrarius *belonged to *B. garinii*, the strain from *R. losea *was identified *as B. afzelii *(Table 1). Table 2 shows the results of our identification of *Borrelia *species by 5S-23S rRNA intergenic spacer-RFLP analysis.

**Table 2:** RFLP analysis of 5S-23S rRNA intergenic spacer and reactivity with mAbs

Strain(s)	Taxon(a)	Source	5S-23S rRNA intergenic spacer	
			
			Amplicon	*MseI *Pattern (band positions [bp])
ZGS01	*B. garinii*	*A.agrarius*	253	B 107,95,51
ZGS02	*B. garinii*	*A.agrarius*	255	C 107,57,51,38
ZGS03	*B. garinii*	*A.agrarius*	253	B 107,95,51
ZGS04	*B. afzelii*	*R. losea*	246	D 107,68,51,20

## Discussion

It has been reported that the primary reservoir hosts in hyperendemic foci of the spirochete in the northeastern and southwestern China are *Apodemus agrarius *and *Clethrionomys rufocanus *[9]. However, information concerning the epidemic status of the disease in western part of China is inadequate. Gansu Province is located in northwestern China, in the midway along the old Silk Road, and has been identified as natural focus of Lyme disease as early as in 1994 [10,11]. In our study we identified two rodent species, *A. agrarius *and *R. losea *harbored *B. burgdorferii *in nature. The high prevalence of *B. burgdorferi *s.l. infection in rodents indicates that an enzootic transmission cycle of *B.burgdorferi *s.l. still exist. Therefore it is important to identify the main local vector tick species responsible for transmission of the Lyme spirochete to humans in future work.

To identify the main reservoir host species in each particular geographic area is important, because the reservoir host species compositon may affect genospecies of *B. burgdorferi *s.l. There are several common characteristics for an efficient reservoir hosts of *B. burgdorferi *s.l. They are abundant in nature, they could naturally infected the *B. burgdorferi *s.l. and remain infective for long periods of time, often for life [12]. In our study we found *A. agrarius *was one of most frequently trapped rodent species and field survey showed the number of *A. agrarius *was huge, they could easily be observed in field and in home. The strains were isolated not only from adult *A. agrarius *but from immature *A. agrarius*, the data suggested the role of *A. agrarius *as the primary reservoir of *B. burgdorferi *s.l. in Gansu Province. As we have mentioned above that *A. agrarius *are distributed over an extensive area in mainland China, and are known to be major reservoir host for *B. burgdorferi *s.l. in China [9]. Combing these data make us believe that *A. agrarius *is a major reservoir host in Gansu Province.

One of the remarkable discoveries of this research was that we firstly isolated *B. burgdorferi *s.l. from *R. losea*, which showed the potential role of *R. losea *in Lyme disease epidemiology in Gansu Province. In fact, previous studies have showed the prevalence of *B. burgdorferi *in *R. losea *(8%) collected in south-east China [13]. However, due to the limited number of *R. losea *in the present study, it is still too early to state that *R. losea *be a reservoir host of *B. burgdorferi *s.l.. It is also unclear whether this rodent could survive long enough for ticks feeding or the agent in rodent remain infectious for ticks. More samples should be collected and the role of this rodent as a source of *B. burgdorferi *s.l. infection for immature ticks should be documented in the future.

In our study three isolates from *A. agrarius *were identified as *B. garinii *and the isolate from *R. losea *was identified as *B. afzelii *based on *Mse*I RFLP analysis. It seemed that there was some specificity between the rodent species and *B.burgdorferi *s.l. genospecies. More samples should be included to illuminate whether there are differences in various genospecies among host ranges.

## Conclusion

The study showed the role of two rodent species in maintaining the pathogen of Lyme disease in the environment from Gansu Province. The isolates which isolated from rodents were identified as two different genospecies.

## Methods

### Rodents collection

**Figure 1:**
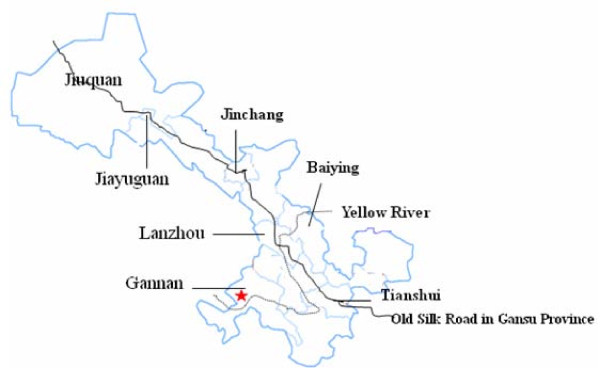
**Study area in Gansu Province**. The black solid line is old silk road in Gansu Province; the dotted line is the Yellow River; pentagon is study area.

During the September and November of 1998, rodents were bait-captured using snap traps in Gannan Tibetan Autonomou Prefecture of Gansu Province which located 420 km south of Lanzhou City (Figure 1). The study area belonged to Diebu forested region, which located on the eastern border of Qinghai-Tibet Plateau, with an elevation of 1 600-4 920 m. The study area mainly are bush grassland and forest grassland with an average elevation of 1600 m (33°40' N, 103°47' E). The temperature ranges from -10 to 25°C, with an average of 6.7°C

### DNA sample preparation

After species identification of the captured rodents, a small piece of spleen was triturated in 2 ml of TE buffer for culture and PCR. After centrifugation, the samples were subjected to DNA extraction using DNA extraction Kit (Sangon) according instruction. DNA of culture isolates were extracted by boiling method. Briefly, cultures were harvested by centrifugation (10,000 × g; 20 min). The bacterial pellet was washed in phosphate-buffered saline and resuspended. The DNA was extracted from the centrifugation pellet of cultivated isolates by boiling in water at 100°C for 10 min, and stored at -20°C until use.

### Culture and identification

The samples from spleen were cultured in 4 ml BSKII medium (Sigma, St Louis, MO, USA) supplemented with 6% rabbit serum and 1% antibiotic mixture for *Borrelia *(Sigma, St Louis, MO, USA) at 32°C. Cultures were subsequently examined for spirochetes by dark-field microscopy for 6 weeks at ×400. Spirochetal isolates were analyzed by IFA with monoclonal antibody. The monoclonal antibody H5332, FITC-labeled goat anti-mouse IgG were friendly provided by Professor Chenxu Ai from Beijing Institute of Microbiology and Epidemiology. The IFA was performed briefly as follow: cultures were harvested by centrifugation and washed three times by suspension in 500 ul of phosphate-buffered saline (PBS) (0.01 M, pH 7.38), recentrifugation at 12,000 × *g *for 25 s, and removal of the supernatant. After being washed, the pellet was resuspended in PBS to a final concentration of 5 × 10^7^/ml. Ten microliters of this suspension was applied to wells on a glass slide. Slides were air dried, fixed in acetone for 10 min, and stored in airtight containers until use. The primary antibody H5332 were diluted from 1:32 to 1:1024 in PBS and then added to wells of slides, and incubated at 37°C for 40 min. After washing, FITC-labeled goat anti-mouse IgG was added at a dilution of 1:20 amd incubated at 37°C for 40 min. After washing, the sildes were examinated by fluorescence microscopy.

### PCR

A nested PCR was performed with primers designed to amplify the variable spacer between two conserved structures, the 3' end of the 5S rRNA and the 5' end of the 23S rRNA as described [14,15]. To minimize contamination, DNA extraction, the reagent setup, amplification and agarose gel electrophoresis were performed in separate rooms.

### RFLP analysis

The culture isolates were further analysed by RFLP to identify their genotypes as described [15,16]. For each one, 13 μl. amplified DNA was digested at 37°C overnight with endonuclease *MseI *(New England Biolabs) according to the manufacturer's recommendations. Electrophoresis was conducted in 16% polyacrylamide gel at 100 V for 3 h. The gels were silver stained, and bands were subsequently visualized under white light. A 50 bp DNA Ladder Marker (TaKaRa, Shuzo) was used as a molecular mass marker. Positive controls of *B. garinii*, *B. afzelii *and *B. burgdorferi s.s*. were prepared in the same way. Genospecies of culture isolates were identified according to RFLP profiles of each sample. RFLP profiles that differed from the known profiles of positive controls were further analysed by sequence analysis.

### DNA sequencing of PCR products

PCR products were purified by using the Qiaquick Gel Extraction kit (Qiagen). The nucleotide sequences were determined by a dideoxynucleotide cycle sequencing method with an automated DNA sequencer (ABI Prism 377, Perkin-Elmer). The sequences obtained in the present study were deposited in GenBank. *MseI *RFLP analysis of the 5S-23S rRNA intergenic spacer was performed on the basis of the DNA sequences obtained using software Vector NTI 9.0 (Lu & Moriyama, 2004).

### Nucleotide sequence accession numbers

The accession numbers of the 5S-23S rRNA intergenic spacer sequences of culture isolates in this study are GQ369934--37.

## Authors' contributions

FZ carried out the samples detection, RFLP analysis and drafted the manuscript. ZJL participated in the design of the study and samples collection. ZWG and JJZ participated in sampling. All authors read and approved the final manuscript.
